# Islamic Attitudes and Rhinoplasty

**Published:** 2018-03

**Authors:** Mehdi Bakhshaee, Masoud Asghari, Mohammad Reza Sharifian, Sogol Jafari Ashtiyani, Bashir Rasoulian

**Affiliations:** 1 *Sinus and Surgical Endoscopic Research Center, Faculty of Medicine, Mashhad University of Medical Sciences, Mashhad, Iran.*; 2 *Department of Otorhinolaryngology-Head and Neck surgery, Faculty of Medicine, Birjand University of Medical Sciences, Birjand, Iran.*; 3 *Student of Pharmacology, International Pardis Pharmacology University of Tehran, Tehran, Iran.*

## Abstract

**Introduction:**

Although the psychological aspects of rhinoplasty have been fully investigated in the medical literature, the religiosity of rhinoplasty candidates has not been taken into consideration.

**Materials and Methods:**

In this cross-sectional study, the religious attitudes of 157 rhinoplasty candidates were compared with those of 74 subjects who had not requested rhinoplasty. A domestic validated reliable questionnaire was completed by all subjects to classify them with respect to religious attitude. Other factors such as age, sex and economic and educational status were also taken into consideration. From the surgeon’s perspective, subjects were put into three categories: subjects who had a relative indication for rhinoplasty (Category.1), subjects with a well-defined nose based on accepted standards of facial aesthetic analysis (Category.2) and finally subjects with a severely deformed nose, such as deviated nose or nasal cleft lip deformity (Category.3).

**Results:**

The mean age among subjects was 28.63 ± 7.05 years, and the majority were female (87%). The two groups of participants (those who did and did not express a desire for rhinoplasty) were analyzed from the point view of age, sex, economic and educational status. The economic and educational status of the two groups did not differ significantly (P>0.05). The religious score showed a significant difference between those who were interested in rhinoplasty (122.75±23.49) and those were not interested (138.78±21.85; P<0.001).

**Conclusion:**

Religion may affect a patient’s decision to undergo rhinoplasty surgery, such that persons with a higher religious attitude tend to undertake it less often. However, individuals with major nasal deformities tend to decide undertake the surgery, irrespective of religious beliefs.

## Introduction

The importance of the aesthetic appeal of the face is the oldest recognized and probably most predictive factor in social relations. The social effects of our appearance begin at birth and exist in every aspect of our lives, whether it is school, family or dating (1-6). For example, a well-proportioned nose might encourage people to think you are more honest, trustworthy, successful and loyal (7). The patient’s motivation for undergoing nasal surgery is multifactorial, even in terms of purely cosmetic surgery, and factors such as socioeconomic, psychological and cultural factors, personality and religious beliefs might impact on the final decision. While the psychological aspects of rhinoplasty have been discussed in several studies (8-13), it is surprising that sociological, cultural and religious factors are almost completely ignored in the literature, with the exception of a few studies (14-17). Indeed, there is still no consensus on the motivations that lead a healthy individual to consider undergoing a cosmetic operation, and it is likely that these motivations vary between cultures.

Consideration of religious or cultural backgrounds is recommended by medical ethicists, even in individuals considered suitable candidates for rhinoplasty (18). 

Furthermore, surgeons should be aware of different perceptions from varying religious, sociocultural and linguistic backgrounds (19-22). Although Jewish and Christian medical ethics are the major reference points in this regard, Islamic medical ethics have not been discussed widely (18). However, in any society, religion exerts a powerful influence on personal perceptions, attitudes and beliefs concerning health care, and shapes the models of care that patients receive (19,23,24).The aim of this study was to investigate the role of a religious attitude as a determining factor for Islamic belief, in the decision to perform rhinoplasty in a group of women from Iran.

## Materials and Methods

*Participants*

This study was conducted at a university and in private practice settings in Mashhad, Iran. The study was permitted by the Institutional Review Board of Mashhad University of Medical Sciences. The study protocol conformed to the ethical guidelines of the Declaration of Helsinki, and personal data

were kept completely secret. In a cross-sectional study, a total of 231 cases were evaluated in terms of religious attitude and desire for rhinoplasty. The subjects were divided into two groups according to a key question “Do you wish to undergo rhinoplasty?” Those who answered “Yes” to the question were assigned to the case group and those who answered “No” were assigned to the control group.

Each patient answered an author-designed questionnaire that asked about their social, economic and educational status. Furthermore, subjects were asked about their religious attitude in order to assess their real commitment to Islamic values.

*Rhinoplasty*

Participants were asked to answer “Yes” or “No” to the question “Have you ever wished to have a cost-free cosmetic rhinoplasty operation?” We used the term “cost-free” because the purpose of the current study was to establish the prevalence of all requests for rhinoplasty without any consideration of economic problems.

*Economic status*

Economic status was measured by self-reported monthly income and adjusted by family size. Subjects were divided into three groups based on their income according to government-defined poverty levels as follows: (1) Low income, which included those earning less than the poverty level; (2) Moderate income, which included those earning up to five times the poverty level; (3) and High income, which included those with an income of more than five times the poverty level.

*Educational status*

Subjects were divided into three categories depending on educational level: (1) Low, completion of some high school education; (2) Moderate, graduation from high school; (3) High, completion of a bachelor’s degree.

*Religious insight*

Two major Islamic practices that might indicate the strength of a person’s religious beliefs are praying and fasting. However, it is doubtful that they are the real indicator for the level of religiosity. Therefore, a validated reliable questionnaire was completed by all subjects in order to classify them with respect to religious attitude (25).

Religious attitude assessments were performed in all participants using a domestic validated questionnaire. The religious attitude questionnaire (in an Islamic tradition) is a 40-item self-report questionnaire which evaluates the religiosity of people. The questions are a closed set according to a five-point Likert-type scale. The answers are scored from 0 to 4; in which a total score of over 100 strongly reveals a higher religious attitude (25).

*Surgical indications*

From the surgeons’ perspective, the participants were put into three categories: subjects with a relative indication for rhinoplasty in whom the criteria for the next two groups were not met (Category.1), subjects with a well-defined nose based on accepted standards of facial aesthetic analysis (Category.2) and finally those with a severely deformed nose, such as a deviated nose or nasal cleft lip deformity (Category.3).

***Statistical analysis***

Data were analyzed using SPSS version 11.5 (SPSS Inc., Chicago, USA). The normality of all variables was assessed by examining their normal plots and using the Kolmogorov-Smirnov test. The Kolmogorov-Smirnov test for quantities data distribution was performed and showed that age did not have normal distribution (P=0.000), but religious attitude score had a normal distribution (P=0.072). Therefore, age was assessed using a nonparametric test (Mann-Whitney U) and religious score was assessed by the T-test. The Pearson Chi-square test was used for data qualities including sex, educational and economic status. P-values less than 0.05 were taken as statistically significant.

## Results

A total of 231 cases participated in the study, the majority of whom were female (87%). The mean age of the subjects was 28.63±7.05 years, ranging between 17 and 55 years. Of these, 157 cases had a tendency toward rhinoplasty and 74 subjects were not interested in undergoing this procedure.

The two groups of participants, those who did and did not desire rhinoplasty, were analyzed from the point view of age, sex and economic and educational status. The economic and educational status of the two groups did not differ significantly (P>0.05) (Table.1).

**Table 1:** The relation between Demographic data/religious attitude score and desire to rhinoplasty.

			**Interested**	**Not Interested**	**Total**	**P Value**
Age		Median(Min-Max)	25(17-55)	30 (18-50)	28(17-55)	
		Religious Score(Mean)	122.75±23.50	138.78±	127.90±24.13	<0.001
Sex	Male	Number (%)	19(12.1)	11(14.9)	30(13.0)	0.560
	Female	Number (%)	138(87.9)	63(85.1)	201(87.0)	
Education	Under Diploma	Number (%)	7(4.5)	11(14.9)	18(7.8)	
	Diploma & College	Number (%)	91(58.0)	26(35.1)	117(50.6)	0.461
	University	Number (%)	59(37.6)	37(50.0)	96(41.6)	
Economy	Poor	Number (%)	12(7.6)	6((8.1)	18(7.8)	
	Average	Number (%)	57(36.3)	30(40.5)	87(37.7)	0.529
	well	Number (%)	88(56.1)	38(51.4)	126(54.5)	
Indication	No	Number (%)	8(5.1)	25(33.8)	33(14.3)	
	Relative	Number (%)	122(77.7)	41(55.4)	163(70.6)	<0.001
	Absolute	Number (%)	27(17.2)	8(10.8)	35(15.2)	

**Fig. 1:**
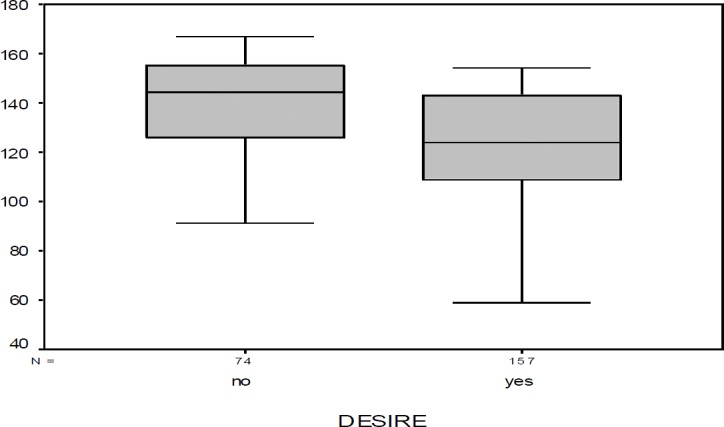
Religious attitude score distribution between those interested in rhinoplasty (desire) and those who were not (no desire)

The religious score showed a significant difference between those who were interested in rhinoplasty (122.75±23.49) and those who were not interested (138.78±21.85; P<0.001) (Fig.1).

To control for confounding factors, we performed univariate and multivariate analysis, and after restriction of the indication we also analyzed data according to relative cases through a multivariate analysis to assess the effect of age and score on the desire for rhinoplasty. 

Again, the score showed a significant association on desire for rhinoplasty (P<0.001; odd ratio [OR]: 0.96 [0.95–0.98]) (Table.2).

**Table 2:** Univariant and multivariate analysis of data for religious attitude

				**Univariant**			**Multivariate**
**Factor**		**β**	**P**	**OR(95%CI)**	**β**	**P**	**OR(95%CI)**
Age		-0.06	0.001	0.94(0.91-0.98)	-0.044	0.032	0.95(0.91-0.99)
Score		-0.36	<0.001	0.96(0.95-0.98)	-0.041	<0.001	0.96( 0.94-0.97)
Indication	No	-	<0.001	1	-	<0.001	1
	Relative	2.35	<0.001	10.54(3.43-32.35)	2.65	<0.001	14.24(4.04-50.17)
	Absolute	2.23	<0.001	9.29(3.89-22.29)	2.73	<0.001	15.33(5.41-43.45)
Constant		-	-	-	5.10	<0.001	170.8

## Discussion

Over the decades, explanations for the motivation for cosmetic surgery have evolved. Initially, internal psychologic conflicts were suggested as a motivation for surgery (26-29). In parallel with the popularity of cosmetic procedures, requesting rhinoplasty is no longer associated with negative psychological connotations, although cynical reports still exist (30-32). Currently, the view is that the patient’s dissatisfaction with their body image persuades them to undergo cosmetic surgery (14,15,33,34). However, it is likely that the factors that motivate a healthy person to undergo an operation are more complex. In particular, in recent years, interest in understanding the effects of religion on health has grown in the medical and scientific communities (35). The principles of most religions, including Islam, profoundly affect human behavior and sentiment and command various strict rules regarding critical health issues (36). Today, it may seem natural that there should be little opposition to cosmetic surgery (37,38); however, up to the start of the 20th century, most religions were opposed to any form of surgery for purely cosmetic reasons. Medical problems and physical disfigurement were considered forms of divine will, and it was not legitimate to correct what God had decreed (38). Although this view may have softened somewhat, studies clearly show that many patients consider religion to be very important and are interested in integrating their religious beliefs into their health care (39).

According to previous studies, females who opted to wear less than the full Islamic veil were significantly more motivated to undergo rhinoplasty compared with those who wore the complete veil (17,40). In addition, those who committed themselves to pray and fast had a significantly reduced tendency toward rhinoplasty compared with other groups (40). However, the lack of accurate information about the actual level of religious attitude is a shortcoming of the medical literature. Although previous studies have assessed some of the more objective and important Islamic practices, including the level of belief in praying, fasting and veil wearing, and their role in the desire to undergo rhinoplasty, this is not the whole story of the Islamic religion. It is possible that the levels of veil wearing, praying and fasting were not exactly correlated with levels of religiosity in former studies. In the current study, we separately assessed the relationship between the level of religious attitude and motivation for rhinoplasty. In the category of patients who had a relative indication for rhinoplasty, the presence of strong religious beliefs was related to a lower demand for surgery. On the other hand, the religion did not show any effect on the opinion of subjects with a severe deformity who had an absolute indication for cosmetic surgery. The findings of our study are in accordance with Islamic law. Although various aspects of Islamic doctrine about cosmetic surgery remain vague, religious scholars generally state that beautification used to improve a deformed part of the body is generally permissible and can eliminate physical and psychological distress. However, manipulating the body for decadent purposes only is not permissible and is therefore unlawful (16). Based on these Islamic beliefs, the reduction of desire to rhinoplasty in subjects with a higher level of religious attitude can be justified.

As mentioned by former studies, body dissatisfaction is the most prominent reason for requesting cosmetic surgery among women. A few reports that assessed body dissatisfaction and religious ideology concluded that Islamic beliefs have a positive impact on body respect (17,41). The effect of some religious practices on body image has been described in several other studies (42-44). 

Here, we conclude that the attitude of women who have stronger religious beliefs was more likely to promote the elevation of spiritual values and internal beauty; while females with less religious attitudes overestimated the importance of the public view of slimness and appearance. These findings can explain the lower demand for undue surgery among religious women.

In our study we controlled for some of the probable factors that might motivate someone toward cosmetic surgery; factors such as educational level, income and some aspects of personality such as hobbies. The two groups who were evaluated were comparable from the point of their income, educational level and hobbies. However, the effect of media exposure was ignored in the study; today, there is increasing sentiment for a thin-ideal in advertising, and therefore media exposure should be considered as an important motivating factor for undergoing cosmetic surgery (45).

## Conclusion

Religion may affect the decision of individuals to undergo rhinoplasty surgery, such that persons with a greater religious attitude tend to do it less often. However, individuals with major nasal deformities may decide to do the surgery irrespective of religious belief.
